# Glycosyltransferase ST6Gal-I promotes the epithelial to mesenchymal transition in pancreatic cancer cells

**DOI:** 10.1074/jbc.RA120.014126

**Published:** 2020-11-23

**Authors:** Colleen M. Britain, Nikita Bhalerao, Austin D. Silva, Asmi Chakraborty, Donald J. Buchsbaum, Michael R. Crowley, David K. Crossman, Yvonne J.K. Edwards, Susan L. Bellis

**Affiliations:** 1 Department of Cell, Developmental, and Integrative Biology, University of Alabama at Birmingham, Birmingham, Alabama, USA; 2 Department of Radiation Oncology, University of Alabama at Birmingham, Birmingham, Alabama, USA; 3 Department of Genetics, University of Alabama at Birmingham, Birmingham, Alabama, USA; 4 Department of Biochemistry and Molecular Genetics, University of Alabama at Birmingham, Birmingham, Alabama, USA

**Keywords:** glycosylation, EGFR, EMT, pancreatic cancer, metastasis, sialic acid, ST6Gal-I, CSC, cancer stem cell, EGFR, epidermal growth factor receptor, EMT, epithelial to mesenchymal transition, EV, empty vector, GSEA, Gene Set Enrichment Analysis, IPA, Ingenuity Pathway Analysis, KD, knockdown, OE, overexpression, PDAC, pancreatic ductal adenocarcinoma, SNA, sambucus nigra agglutinin

## Abstract

ST6Gal-I, an enzyme upregulated in numerous malignancies, adds α2-6-linked sialic acids to select membrane receptors, thereby modulating receptor signaling and cell phenotype. In this study, we investigated ST6Gal-I’s role in epithelial to mesenchymal transition (EMT) using the Suit2 pancreatic cancer cell line, which has low endogenous ST6Gal-I and limited metastatic potential, along with two metastatic Suit2-derived subclones, S2-013 and S2-LM7AA, which have upregulated ST6Gal-I. RNA-Seq results suggested that the metastatic subclones had greater activation of EMT-related gene networks than parental Suit2 cells, and forced overexpression of ST6Gal-I in the Suit2 line was sufficient to activate EMT pathways. Accordingly, we evaluated expression of EMT markers and cell invasiveness (a key phenotypic feature of EMT) in Suit2 cells with or without ST6Gal-I overexpression, as well as S2-013 and S2-LM7AA cells with or without ST6Gal-I knockdown. Cells with high ST6Gal-I expression displayed enrichment in mesenchymal markers (N-cadherin, slug, snail, fibronectin) and cell invasiveness, relative to ST6Gal-I-low cells. Contrarily, epithelial markers (E-cadherin, occludin) were suppressed in ST6Gal-I-high cells. To gain mechanistic insight into ST6Gal-I’s role in EMT, we examined the activity of epidermal growth factor receptor (EGFR), a known EMT driver. ST6Gal-I-high cells had greater α2-6 sialylation and activation of EGFR than ST6Gal-I-low cells. The EGFR inhibitor, erlotinib, neutralized ST6Gal-I-dependent differences in EGFR activation, mesenchymal marker expression, and invasiveness in Suit2 and S2-LM7AA, but not S2-013, lines. Collectively, these results advance our understanding of ST6Gal-I’s tumor-promoting function by highlighting a role for ST6Gal-I in EMT, which may be mediated, at least in part, by α2-6-sialylated EGFR.

Pancreatic ductal adenocarcinoma (PDAC) remains one of the most lethal malignancies, with a dismal 5-year survival rate of less than 9% (https://www.cancer.org/cancer/pancreatic-cancer/detection-diagnosis-staging/survival-rates.html, accessed September 1, 2020). Most patients are diagnosed after metastatic lesions have formed, making treatment difficult and often unsuccessful. It is thought that PDAC metastasizes early during the carcinogenic process owing to the emergence of stem-like cancer cells with high metastatic potential, termed cancer stem cells (CSCs) (1). CSCs are inherently invasive and apoptosis resistant, and hence, these cells are major drivers of cancer progression (2). Considerable efforts have been aimed at elucidating the functional role of stem-like cancer cells in metastasis; however, the contribution of the cellular glycome to this process has received insufficient attention. Recent studies have suggested that the glycosyltransferase, ST6Gal-I, promotes CSC characteristics (3) and acts as a survival factor to protect cells against cytotoxic assaults including chemotherapy (4, 5, 6), radiation (7), serum deprivation (8), and hypoxia (9). ST6Gal-I is a sialyltransferase that adds an α2-6-linked sialic acid to *N*-glycosylated proteins that are destined for the plasma membrane or secretion. ST6Gal-I is upregulated in multiple cancers, including ovarian, pancreatic, and colon (3, 10, 11, 12, 13), and high expression of this enzyme correlates with a poor patient prognosis (3, 11, 12, 13). Interestingly, ST6Gal-I expression is induced by oncogenic Ras signaling (14). Activating mutations in K-Ras are found in more than 90% of patients with PDAC (15), and these mutant isoforms appear early in tumor development, as indicated by their presence in the premalignant lesions, PanINs (16). Likewise, ST6Gal-I is strongly expressed in PanINs, whereas normal pancreatic acinar cells lack detectable ST6Gal-I protein expression (3).

An enrichment in tumor cell sialylation has long been implicated in neoplastic transformation and tumor-promoting cellular behaviors such as invasiveness and apoptosis resistance (17, 18). The addition of sialic acid to membrane receptors can profoundly affect cell signaling and phenotype owing to sialylation-dependent changes in receptor features such as conformation, oligomerization, and/or cell surface retention. In particular, the α2-6 sialic acid linkage is often increased upon malignant transformation (19) and is similarly elevated in certain nonmalignant stem cell populations (20). As ST6Gal-I is the predominant enzyme responsible for the α2-6 sialylation of *N*-glycosylated proteins, understanding its function in cancer is critical. ST6Gal-I imparts a malignant cell phenotype by regulating, *via* sialylation, key receptors that control tumor-associated signaling networks. For example, ST6Gal-I-mediated sialylation of the β1 integrin promotes cell migration and invasion (21, 22), whereas α2-6 sialylation of the Fas and tumor necrosis factor receptor 1 death receptors inhibits apoptosis by hindering receptor internalization (23, 24). Furthermore, our group recently determined that sialylation of epidermal growth factor receptor (EGFR) by ST6Gal-I enhances both basal and ligand-dependent EGFR activation and protects against gefitinib-induced apoptosis (25).

EGFR is a receptor tyrosine kinase with a rich history in cancer pathogenesis. EGFR is heavily glycosylated, and it is well known that the *N*-glycans play a major part in modulating EGFR structure and function (26, 27, 28). The sialylation of EGFR can influence ligand binding, receptor clustering, and, consequently, downstream signaling (29, 30). The activation of EGFR elicits a variety of biological outcomes, including cell proliferation, the cell’s response to DNA damage, and the epithelial to mesenchymal transition (EMT) (31, 32, 33). As with CSCs, cancer cells undergoing EMT reactivate developmental pathways that facilitate invasiveness and apoptosis resistance. In fact, EMT has been proposed as a major mechanism responsible for generating CSCs (34). A role for ST6Gal-I in TGFβ-driven EMT has been previously reported. Gu’s group found that ST6Gal-I was selectively upregulated by TGFβ in the GE11 mouse epithelial cell model, and importantly, ST6Gal-I activity was required for TGFβ-stimulated EMT (35). However, the contribution of ST6Gal-I to EGFR-mediated EMT has not previously been investigated. Given the relationship between EGFR and EMT, combined with the finding that ST6Gal-I enhances EGFR activation, we interrogated whether α2-6 sialylation of EGFR promotes EMT in PDAC cells.

The Suit2 PDAC cell line and its isogenic, metastatic subclones, S2-013 and S2-LM7AA, were used to delineate the role of ST6Gal-I in EMT. Notably, ST6Gal-I is markedly upregulated in the metastatic subclones as compared with parental Suit2 cells. RNA-Seq experiments highlighted EMT, cell motility, and stem cell–associated gene networks as prominent pathways upregulated in the metastatic lines. Supporting a functional role for ST6Gal-I in the metastatic phenotype, forced expression of ST6Gal-I in the parental, poorly metastatic Suit2 line (with low endogenous ST6Gal-I) conferred a phenotype similar to that of the metastatic subclones, evidenced by enrichment in EMT, stem cell, and cell motility networks. To further establish a role for ST6Gal-I in EMT, ST6Gal-I expression was knocked down in the metastatic lines, complementing the overexpression of ST6Gal-I in the parental line. In all three of the cell models, high expression of ST6Gal-I led to increased α2-6 sialylation and activation of EGFR, as well as upregulated expression of mesenchymal markers and cell invasiveness. Moreover, the EGFR inhibitor, erlotinib, neutralized ST6Gal-I-dependent differences in EGFR sialylation and activation, EMT marker expression, and invasiveness in Suit2 and S2-LM7AA cells. In the aggregate, these results highlight the importance of ST6Gal-I activity in driving EMT, a critical process that promotes metastatic disease.

## Results

### ST6Gal-I’s contribution to activation of gene networks that promote a metastatic phenotype

**Figure 1:**
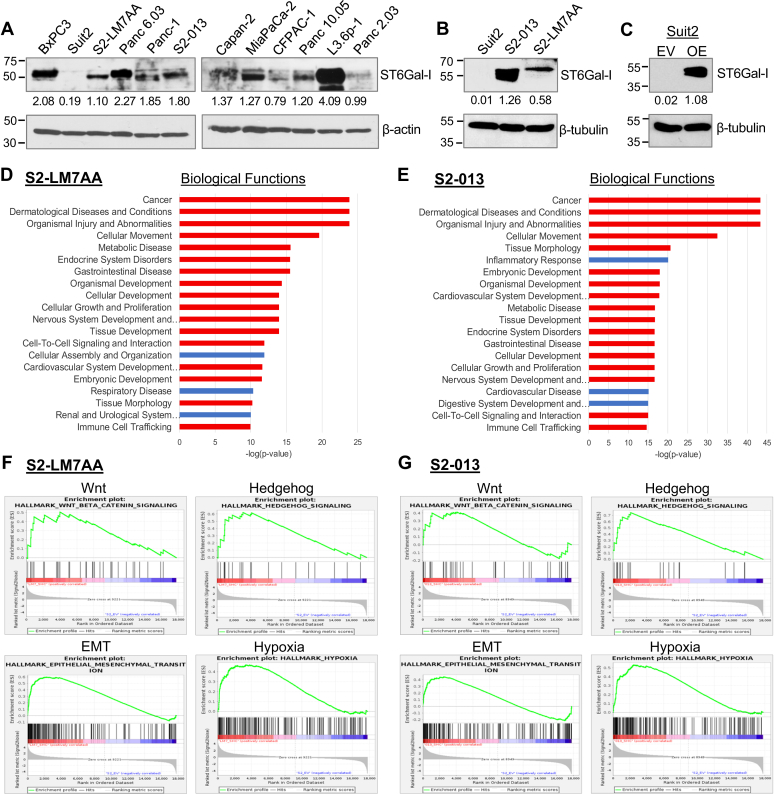
**ST6Gal-I is upregulated in S2-LM7AA and S2-013 metastatic subclones, which display enrichment in gene networks associated with stemness and EMT**. *A*, ST6Gal-I is expressed in most human PDAC cell lines, with the exception of Suit2 cells. *B*, ST6Gal-I is upregulated in Suit2-derived metastatic subclones, S2-013 and S2-LM7AA. *C*, ST6Gal-I was overexpressed (OE) in parental Suit2 cells. Control lines were transduced with an empty vector (EV). Both lines represent stable, polyclonal populations. Densitometric values in *A–C* were normalized to their respective loading controls. *D–E*, RNA-Seq data collected from S2-LM7AA (*D*) and S2-013 (*E*) lines were compared with data from Suit2 EV cells. The top 20 Biological Functions altered in S2-LM7AA and S2-013 cells are shown. *Red bars* denote Functions that are shared between the two lines. *F–G*, GSEA of S2–LM7AA (*F*) and S2-013 (*G*) cells relative to Suit2 EV cells revealed enrichment in the Wnt, Hedgehog, EMT, and Hypoxia pathways. The normalized enrichment score (NES) and false discovery rate (FDR) values for S2-LM7AA cells are: Wnt, NES = 1.47; FDR = 0.05; Hedgehog, NES = 1.64; FDR = 0.015; EMT, NES = 2.22; FDR < 0.0005; and Hypoxia, NES = 1.76, FDR = 0.005. For S2-013, the values are: Wnt, NES = 1.18; FDR = 0.33; Hedgehog, NES = 1.95; FDR < 0.0005; EMT, NES = 1.65; FDR = 0.012; and Hypoxia, NES = 2.00, FDR < 0.0005.

Immunoblotting for ST6Gal-I in multiple human PDAC cell lines revealed that, although most lines had considerable ST6Gal-I expression, the Suit2 line had negligible levels of ST6Gal-I (Fig. 1*A*). Suit2 cells have relatively low metastatic potential in animal models, and in order to study the metastatic process, other investigators have developed metastatic subclones of the Suit2 line. For example, the metastatic S2-LM7AA line was generated through iterative *in vivo* selection, yielding a population that reliably metastasizes to the liver following injection into the pancreas (36). A second metastatic subclone, S2-013, metastasizes to the lungs when grown as a subcutaneous tumor (37, 38). Significantly, both of the Suit2-derived metastatic subclones displayed elevated ST6Gal-I expression when compared with the parental Suit2 line (Fig. 1*B*).

RNA-Seq was conducted on the S2-LM7AA and S2-013 subclones to gain insight into metastasis-associated pathways. To determine whether the upregulation of ST6Gal-I in these subclones contributed to metastatic characteristics, RNA-Seq was also performed on parental Suit2 cells with forced ST6Gal-I overexpression (OE) or cells alternatively transduced with an empty vector (EV) construct (Fig. 1*C*). We first compared the RNA-Seq data generated from the two metastatic subclones. Table S1 shows the top 50 upregulated (A) and downregulated (B) genes in the S2-LM7AA and S2-013 lines, as compared with poorly metastatic Suit2 EV cells. RNA-Seq confirmed a 3.5-fold upregulation of ST6Gal-I in S2-LM7AA cells and 16-fold upregulation in S2-013 cells (not shown). Ingenuity Pathway Analysis (IPA) was employed to identify the top 20 Biological Functions altered in the metastatic lines relative to Suit EV cells. As shown in Figure 1, *D* and *E*, a strong correspondence was noted between the two subclones, illustrated by the fact that 17 of 20 of the Biological Functions were shared (denoted by red bars). These included cancer, gastrointestinal disease, developmental pathways (organismal development, tissue development), and cancer-associated cell functions (cellular movement, cellular development, cellular growth and proliferation). Gene Set Enrichment Analysis (GSEA) of the normalized gene expression showed that, compared with Suit2 EV cells, S2-LM7AA and S2-013 cells were enriched in stemness-associated networks (Wnt, Hedgehog), EMT, and processes related to EMT such as hypoxia (Fig. 1, *F* and *G*). We also utilized the IPA Upstream Regulator module to determine that the two metastatic subclones exhibited activation of regulators central to stem cell networks, EMT, cell growth and proliferation, and cell migration (Table S2).

**Figure 2:**
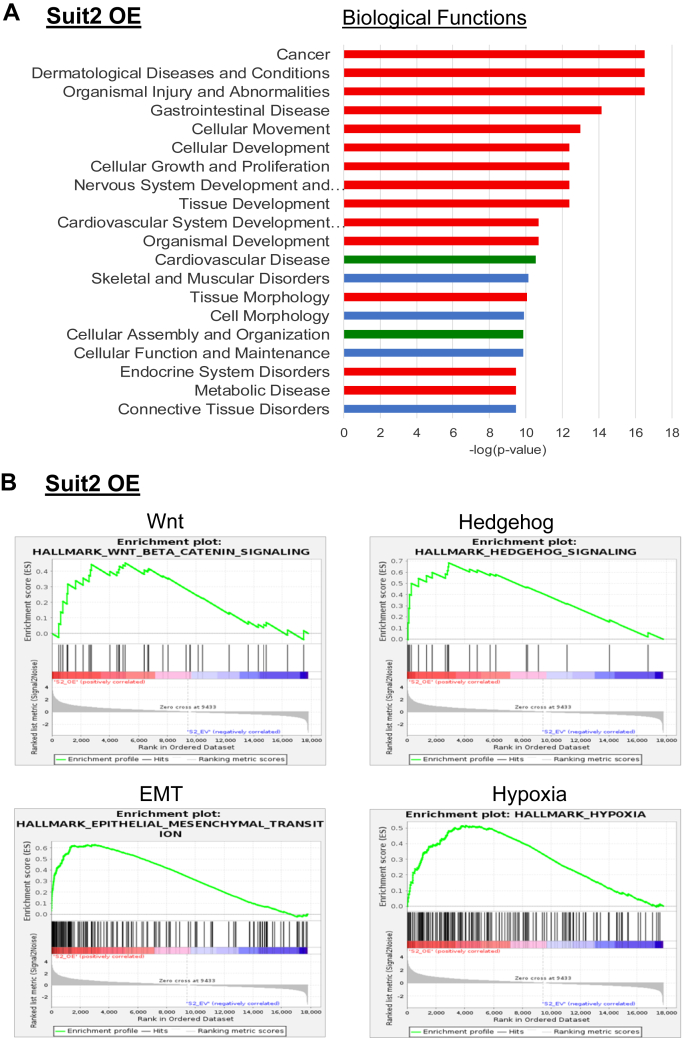
**Overexpression of ST6Gal-I in Suit2 cells promotes activation of metastasis-associated gene networks including stemness and epithelial to mesenchymal transition (EMT) pathways**. *A*, graph depicts the top 20 IPA Biological Functions altered in Suit2 overexpression (OE) cells as compared with Suit2 empty vector (EV) cells. Fourteen of the Biological Functions altered in Suit2 OE cells were also altered in both S2-LM7AA and S2-013 cells (*red bars*). Another two Functions were shared with one of the two subclones (*green bars*). *B*, gene Set Enrichment Analysis of Suit2 OE versus EV cells show that OE cells are enriched in the following pathways: Wnt (normalized enrichment score [NES] = 1.34; false discovery rate [FDR] = 0.10), Hedgehog (NES = 1.84, FDR = 0.001), EMT (NES = 2.24, FDR < 0.0005) and Hypoxia (NES = 1.85, FDR = 0.001).

Given the upregulation of ST6Gal-I observed in the metastatic subclones, we compared RNA-Seq data from ST6Gal-I OE and EV cells to determine whether forced overexpression of ST6Gal-I was sufficient to confer metastatic characteristics. Table S3 shows the top 50 upregulated and downregulated genes in Suit2 OE cells relative to EV cells. An analysis of the top 20 IPA Biological Functions (Fig. 2*A*) revealed that forced expression of ST6Gal-I in Suit2 cells altered 14 of 17 of the pathways that were modulated in both of the metastatic subclones (red bars). Another two of the Functions were common to one of the two metastatic lines (green bars). Functions shared with both of the metastatic subclones included cancer, gastrointestinal disease, developmental pathways (organismal development, tissue development), and tumorigenic cell functions (cellular movement, cellular development, cellular growth and proliferation). GSEA indicated that ST6Gal-I overexpression activated Wnt, Hedgehog, EMT, and hypoxia networks (Fig. 2*B*). In addition, the IPA Upstream Regulators activated in Suit2 OE cells were very similar to those that activated in the metastatic subclones, specifically, regulators involved in EMT, stemness, hypoxia, and cell motility (Table S4). To pinpoint pathways that may be particularly important for metastasis, we identified the Upstream Regulators that were coordinately activated in S2-013 and S2-LM7AA cells (Table 1A). We then compared these Upstream Regulators to those altered in Suit2 OE cells (Table 1A). As shown, about two-thirds (41/63) of the pathways activated in both of the metastatic lines were also activated in Suit2 OE cells relative to Suit2 EV cells. These Upstream Regulators included molecules involved in (i) stemness (WNT3A, β-catenin, LEF1, SHH, JAG1, GLI1, WBP2); (ii) EMT (TGFβ1, SMAD1-4, BMP2, GDF9, GLI1, HIF1A, WWTR1, ROR1, MRTFA, JAK1/2); and (iii) cell migration (ROCK1, RAF1, ARNT2, ROR1, SIM1, TEAD4, WWTR1, MRTFA). Finally, we screened for shared pathways in the IPA Biological Functions and Canonical Pathways databases and observed substantial activation of pathways involved in EMT and cell motility in the three lines with high ST6Gal-I expression, S2-013, S2-LM7AA, and Suit2 OE (Table 1B). Taken together, these data strongly suggest that the forced expression of ST6Gal-I in the parental, poorly metastatic Suit2 line is sufficient to activate many of the metastasis-associated processes upregulated in the metastatic subclones, S2-013 and S2-LM7AA.

**Table 1:** Pathways Shared by Metastatic Subclones and Suit2 OE cells

A. Upstream regulators with predicted activation			
Regulator	S2-013 versus	S2-LM7AA versus	Suit2 OE versus
	Suit2 EV	Suit2 EV	Suit2 EV
	*Z* score	*Z* score	Z score
APLN	2.164	1.554	2.728
ARNT2	3.569	2.335	4
BDNF	2.943	2.914	2.58
BMP2	1.981	1.62	2.381
BRD4	2.273	2.178	2.626
CBX5	3.317	2.414	
CCR2	3.687	3.111	1.89
CG	1.857	2.268	2.287
CIITA	2.27	1.946	1.82
CLEC4G	1.994	2.121	
CTNNB1	2.493	3.043	1.656
D-glucose	2.569	2.861	2.118
DSCAM	4.122	2.683	2.688
DSCAML1	3.452	1.877	3.3
EREG	2.012	1.982	
F2	2.45	3.441	2.787
FBXO32	2.467	1.655	
GDF9	1.74	2.392	1.936
GLI1	2.433	2.787	2.208
Growth hormone	2.226	1.807	2.469
HIF1A	2.695	1.706	3.905
HRG	2.138	2.236	2
IL10	2.115	1.674	
IL6	1.505	2.34	
JAG1	2.315	2.223	3.086
JAK1/2	2.065	1.732	2.433
KDM3A	2.197	2.241	
KMT2D	2.468	1.694	
LATS2	2.534	1.706	
LEF1	2.083	2.314	3.026
LLGL2	2.887	3.317	2.646
MAPK1	3.046	2.852	
MRTFA	1.674	3.491	2.078
MRTFB	3.466	4.182	2.883
MYOD1	1.901	2.166	
NMNAT1	2.197	1.976	
NORAD	2.138	2.714	1.974
NOTCH1	2.842	1.988	
NRG1	3.729	3.789	3.696
NSUN6	3.5	2.887	2.646
PLAG1	2.377	1.912	
progesterone	1.861	2.311	
RAF1	1.979	2.163	2.936
ROCK1	2.178	2.621	2.425
ROR1	2.074	2.813	2.236
RXFP2	1.633	2	
SHH	1.823	2.95	2.689
SIM1	3.637	2.038	3.615
SMAD1	1.676	2.805	2.038
Smad2/3-Smad4	1.698	1.98	3.296
SMAD3	2.796	2.791	2.264
SNAI2	2.343	1.651	
STAT5B	2.336	1.681	
TEAD1	3.979	2.309	
TEAD2	3.3	2.53	
TEAD3	3.578	2.111	
TEAD4	4.086	2.137	2.449
Tgf beta	3.452	4.016	3.543
TGFB1	3.141	4.557	3.943
WBP2	2.419	2.212	3.036
WNT3A	1.954	3.388	3.535
WWTR1	2.697	2.041	1.698
ZEB1	1.822	2.06	

B. Biological functions and canonical pathways			
Regulator	S2-013 versus	S2-LM7AA versus	Suit2 OE versus
	Suit2 EV	Suit2 EV	Suit2 EV
	*Z* score	*Z* score	Z score
Regulation of EMT by growth factors pathway	2.309	2.357	2.137
Regulation of EMT in development pathway	2.333	2.357	2.132
Colorectal cancer metastasis signaling	2.837	2.556	2.188
Ephrin receptor signaling	2.828	2.673	2.414
Integrin signaling	3.464	1.789	2.646
Migration of cells	4.786	3.972	3.622
Migration of tumor cell lines	3.206	1.798	3.64
Microtubule dynamics	6.343	4.302	3.901
Organization of cytoskeleton	6.042	4.267	4.084
Chemotaxis	5.839	3.648	2.004
Formation of cellular protrusions	6.104	3.636	3.881
Cell movement	5.629	4.219	3.315
Cell movement of tumor cell lines	3.552	1.543	2.709

### Cells with high ST6Gal-I expression exhibit enhanced expression of mesenchymal markers

**Figure 3:**
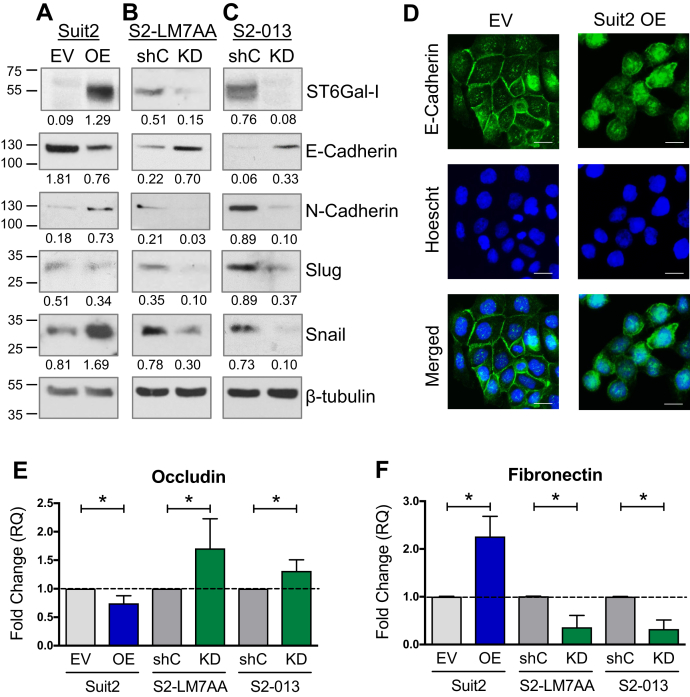
**Cells with high ST6Gal-I expression display a mesenchymal phenotype.***A–C*, to complement the Suit2 overexpression (OE) model (*A*), ST6Gal-I was knocked down (KD) in S2-LM7AA (*B*) and S2-013 (*C*) cells. Cells expressing a nontargeting shRNA were used as the control (shC). High expression of ST6Gal-I in the three cell models was associated with increased expression of mesenchymal markers (N-cadherin, slug, and snail), whereas the epithelial marker (E-cadherin) was decreased in high ST6Gal-I expressers. Densitometric values were normalized to the β-tubulin loading control. *D*, immunocytochemistry for E-cadherin in the Suit2 line shows reduced junctional staining in Suit2 OE cells. The scale bar represents 10 μm. *E–F*, downstream transcriptional targets of slug and snail, occludin (*E*), and fibronectin (*F*) were measured using qRT-PCR. Graphs represent the means ± SEMs from three independent experiments, with each experiment performed in triplicate. ∗denotes *p* < 0.05.

Based on the RNA-Seq results, we further investigated the role of ST6Gal-I in EMT. To supplement the Suit2 OE cell model (Fig. 3*A*), ST6Gal-I was knocked down (KD) in the metastatic S2-LM7AA and S2-013 lines (Fig. 3, *B* and *C*, respectively). A nontargeting shRNA sequence was used as the control (shC). The expression of EMT markers was examined in cells with modulated ST6Gal-I expression. A switch between E-cadherin, an epithelial cadherin, and N-cadherin, a mesenchymal cadherin, is a hallmark of EMT. In addition, cells that have undergone EMT exhibit an upregulation in the mesenchymal transcription factors, slug and snail. As compared with Suit2 EV cells, Suit2 OE cells had increased expression of the mesenchymal markers, snail and N-cadherin, although slug expression was unchanged (Fig. 3*A*). Contrarily, the epithelial marker, E-cadherin, was reduced in OE cells. Consistent with these results, knockdown of ST6Gal-I in the metastatic S2-LM7AA and S2-013 lines strongly suppressed the expression of snail, slug, and N-cadherin, whereas E-cadherin expression was enhanced in KD cells (Fig. 3, *B* and *C*).

An important event in EMT is the breakdown of E-cadherin-containing cell–cell junctions, an event that promotes a more mesenchymal phenotype and facilitates cell motility. E-cadherin localization was examined by immunocytochemistry in Suit2 EV and OE cells. Suit2 EV cells displayed strong E-cadherin localization at the cell–cell junctions, whereas the staining in OE cells was intracellular and not distinctively membrane bound (Fig. 3*D*). Staining of the two metastatic subclones did not yield any overt differences in E-cadherin localization between shC and KD cells; however, there was little junctional staining in any of these lines (data not shown). We hypothesize that, since shC cells are already highly metastatic, knockdown of ST6Gal-I alone was insufficient to restore strong cell–cell junctions (although overall levels of E-cadherin were enhanced by ST6Gal-I KD, Fig. 3, *B* and *C*).

We next assessed downstream transcriptional targets of slug and snail using qRT-PCR. Slug and snail regulate the transcription of numerous genes involved in EMT including occludin, an epithelial marker suppressed during EMT, and fibronectin, a mesenchymal marker upregulated during EMT. In comparison with Suit2 EV cells, OE cells had decreased levels of occludin mRNA (Fig. 3*E*), whereas ST6Gal-I KD in the S2-LM7AA and S2-013 lines caused an increase in occludin expression (Fig. 3*E*). An opposite expression pattern was noted for fibronectin, where fibronectin mRNA levels were augmented in Suit2 cells with forced OE but reduced by ST6Gal-I KD in the two metastatic lines (Fig. 3*F*).

### ST6Gal-I activity increases cellular invasiveness

**Figure 4:**
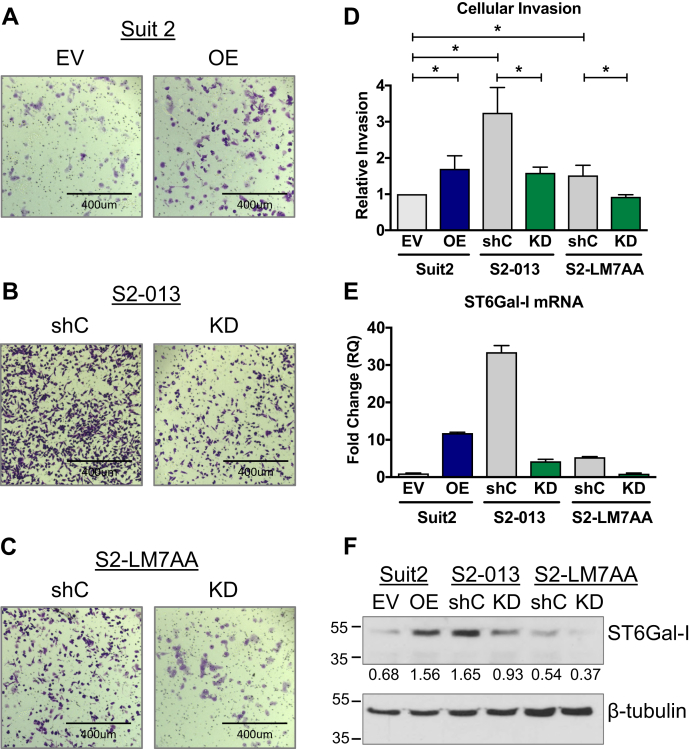
**Cells with high ST6Gal-I expression are more invasive.***A–C*, representative images of transwell invasion assays for Suit2 (*A*), S2-013 (*B*), and S2-LM7AA (*C*) cells. *D*, Cells invading through matrigel-coated transwell filters were stained with crystal violet and then solubilized in 10% acetic acid. Invasion was quantified by absorbance spectroscopy. Absorbance values were normalized to those of controls (cells plated in chambers with no chemoattractant). Graph represents the means ± SEMs from at least three independent experiments. ∗denotes *p* < 0.05. *E*, Representative mRNA expression of ST6Gal-I from at least three independent experiments. *F*, ST6Gal-I protein expression. Densitometric values were normalized to the β-tubulin loading controls.

As cell invasiveness is a seminal phenotypic feature of EMT, invasion assays were conducted using Matrigel-coated transwell chambers. Cells were allowed to invade for 24 h, and cells migrating to the underside of the matrigel-coated filters were stained with crystal violet. Stained cells were solubilized in 10% acetic acid and invasion quantified by absorbance spectroscopy. S2-013 and S2-LM7AA shC cells were inherently more invasive than Suit2 EV cells, consistent with the metastatic phenotype of these two subclones (representative images in Fig. 4, *A*–*C*, quantification in Fig. 4*D*). Upon forced ST6Gal-I OE, Suit2 cells displayed a significant increase in invasion (Fig. 4, *A* and *D*). In contrast, KD of ST6Gal-I suppressed the invasive capabilities of the metastatic S2-013 and S2-LM7AA lines (Fig. 4, *B*–*D*). Interestingly, the overall degree of cell invasiveness was correlated with the levels of ST6Gal-I mRNA (Fig. 4*E*) and protein (Fig. 4*F*). Cells with the highest amount of ST6Gal-I (*e.g.*, S2-013 shC cells) were the most invasive.

### Cells with high ST6Gal-I expression display increased α2-6 sialylation and activation of EGFR

**Figure 5:**
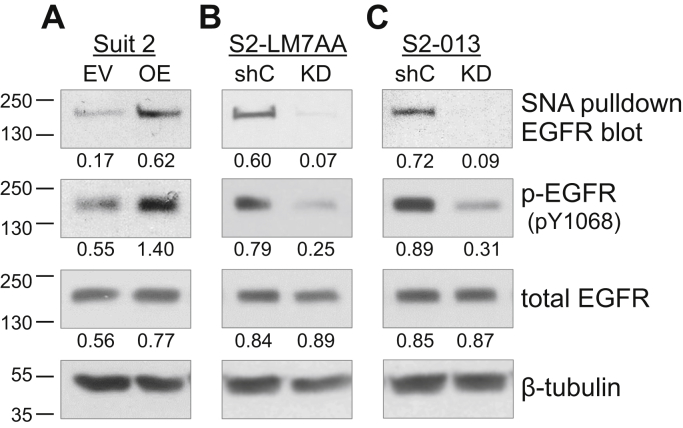
**Cells with high ST6Gal-I expression exhibit increased α2-6 sialylation and activation of EGFR**. Suit2 (*A*), S2-LM7AA (*B*), and S2-013 (*C*) cells with modulated ST6Gal-I expression were evaluated for sialylation and activation of EGFR. ST6Gal-I-sialylated proteins were precipitated using SNA lectin and then immunoblotted for EGFR. To evaluate EGFR activation state, whole-cell lysates were immunoblotted for phosphorylated (pY1068) or total EGFR. Densitometric values were normalized to the β-tubulin loading control.

EGFR is an important driver of EMT, and our prior studies demonstrated that ST6Gal-I-mediated sialylation of EGFR promotes both basal and ligand-dependent EGFR activation in pancreatic and ovarian cancer cells (25). To evaluate EGFR sialylation levels, we conducted pull-down experiments using sambucus nigra agglutinin (SNA), a lectin that selectively binds to the α2-6 sialic acid linkage elaborated by ST6Gal-I. Cell lysates were incubated with agarose-conjugated SNA to precipitate α2-6 sialylated proteins, and then precipitates were immunoblotted for EGFR. Suit2 EV cells, with low endogenous ST6Gal-I, had low levels of α2-6-sialylated EGFR, whereas cells with ST6Gal-I OE displayed enriched sialylation of EGFR (Fig. 5*A*). The total amount of EGFR was comparable in Suit2 EV and OE cells, indicating that manipulation of ST6Gal-I expression did not alter overall levels of EGFR expression. Importantly, the overexpression of ST6Gal-I caused an increase in the basal activation of EGFR, evidenced by immunoblotting for phosphorylated EGFR (Fig. 5*A*). SNA precipitation assays were next conducted with the S2-LM7AA and S2-013 lines. As shown in Fig. 5, *B* and *C*, EGFR was sialylated in S2-LM7AA and S2-013 shC cells; however, levels of α2-6 sialylated EGFR were greatly reduced in the ST6Gal-I KD lines. Knockdown of ST6Gal-I also resulted in a decrease in basal EGFR activation, whereas total levels of EGFR were equivalent in EV and KD cells. These results suggest that α2-6 sialylation of EGFR by ST6Gal-I is sufficient to induce EGFR activation, independent of ligand binding.

### Inhibition of EGFR attenuates the differential effects of ST6Gal-I on EMT marker expression and cell invasiveness

**Figure 6:**
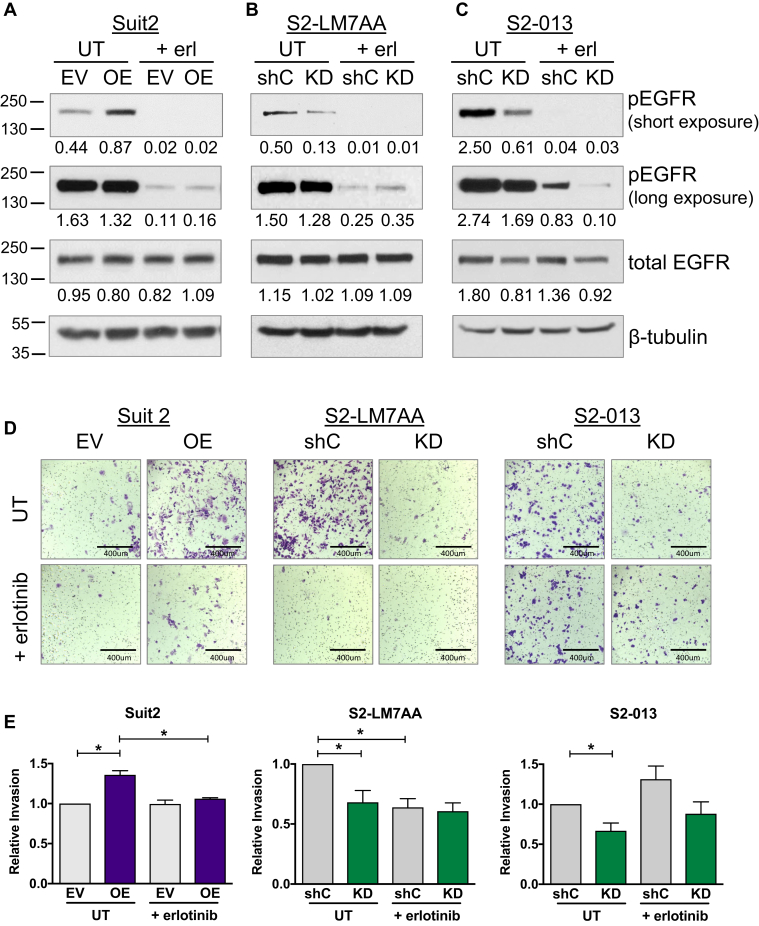
**EGFR inhibition attenuates the invasive phenotype conferred by ST6Gal-I in Suit2 and S2-LM7AA cells**. *A*, Suit2, *B*, S2-LM7AA, and *C*, S2-013 cells were treated for 24 h with erlotinib (+erl) or left untreated (UT). Cell lysates were immunoblotted for phospho and total EGFR. Short and long film exposures are shown for the phospho-EGFR blots. Densitometric values in A–C were normalized to the loading control. *D*, Suit2 parental and metastatic cell lines were treated with or without erlotinib and then subjected to invasion assays. Representative images are shown of crystal violet–stained cells. *E*, crystal violet–stained cells were solubilized with 10% acetic acid, and relative invasion was quantified by absorbance spectroscopy. Absorbance values were normalized to those of controls (cells seeded in chambers with no chemoattractant). Graphs represent the means ± SEMs from at least three independent experiments, with each experiment performed in triplicate. ∗denotes *p* < 0.05.

In view of the enhanced EGFR activation in cells with high ST6Gal-I expression, we evaluated the role of sialylated EGFR in directing EMT. Cells were treated with the EGFR tyrosine kinase inhibitor, erlotinib, and probed for EGFR activation by immunoblotting. As shown in Fig. 6*A*, erlotinib treatment of Suit2 EV and OE cells not only diminished the overall activation of EGFR but also eliminated the ST6Gal-I-dependent differences in EGFR activation (Fig. 6*A*, long exposure). Similarly, ST6Gal-I-mediated differences in EGFR activation were ablated by erlotinib in the S2-LM7AA line (Fig. 6*B*). On the other hand, erlotinib treatment did not eliminate ST6Gal-I-dependent differences in S2-013 cells; shC cells maintained substantially higher levels of activated EGFR than KD cells in the presence of erlotinib, although overall levels of EGFR activation were reduced by the drug in both lines (Fig. 6*C*).

To determine whether inhibiting EGFR suppressed the invasive capabilities imparted by ST6Gal-I, invasion assays were conducted in the presence or absence of erlotinib. As observed previously, untreated Suit2 OE cells were more invasive than untreated EV cells; however, this difference was abolished by erlotinib treatment (representative images in Fig. 6*D*, quantification in Fig. 6*E*). Likewise, the enhanced invasiveness of S2-LM7AA shC cells, as compared with KD cells, was eliminated by erlotinib treatment (Fig. 6, *D* and *E*). However, erlotinib seemed to have little effect on the invasiveness of S2-013 cells (Fig. 6, *D* and *E*). Because S2-013 invasiveness was not inhibited by erlotinib, the role of EGFR in EMT was not examined further in this line.

**Figure 7:**
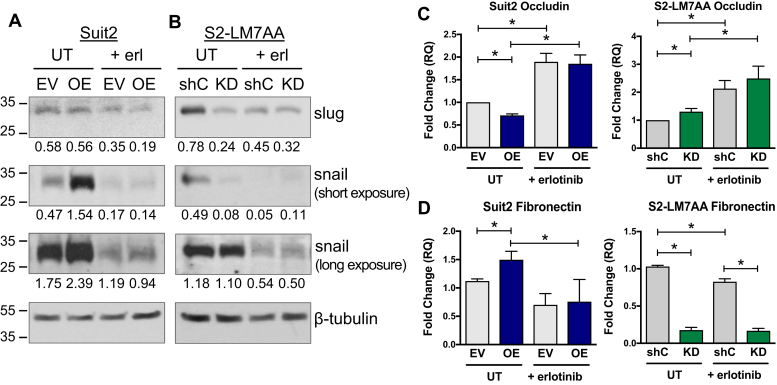
**Inhibition of EGFR suppresses ST6Gal-I’s activity in promoting expression of mesenchymal markers**. *A–B*, immunoblotting for slug and snail in Suit2 (*A*) and S2-LM7AA (*B*) cells treated with or without erlotinib. Densitometric values in *A* and *B* were normalized to the loading control. *C–D*, qRT-PCR analyses of occludin (*C*) and fibronectin (*D*) mRNA expression in cells treated with or without erlotinib. Graphs represent the means ± SEMs from at least three independent experiments, with each experiment performed in triplicate. ∗denotes *p* < 0.05.

The expression of EMT markers was next assessed in Suit2 and S2-LM7AA cells treated with or without erlotinib (Fig. 7, *A* and *B*). Consistent with data in Fig. 3, untreated Suit2 ST6Gal-I OE cells had elevated expression of snail relative to EV cells, with no difference in slug expression. Erlotinib treatment reduced the overall expression of snail and also neutralized the ST6Gal-I-dependent differences in snail expression (Fig. 7*A*, long exposure). In the S2-LM7AA line, untreated shC cells exhibited higher expression of both slug and snail in comparison with ST6Gal-I KD cells. Treatment with erlotinib abrogated ST6Gal-I-dependent differences in expression of these proteins (Fig. 7*B*, long exposure).

We also evaluated the expression of occludin and fibronectin in erlotinib-treated cells. As shown previously, untreated cells with high ST6Gal-I expression (Suit2 OE and S2-LM7AA shC) had diminished occludin levels in comparison with cells with low ST6Gal-I expression (Suit2 EV and S2-LM7AA KD) (Fig. 7*C*). Interestingly, erlotinib treatment increased the expression of occludin in all of the cell lines, consistent with the concept that blocking EGFR activity promotes a more epithelial phenotype. Furthermore, the differential effects of ST6Gal-I activity on occludin expression were abolished by erlotinib treatment in the Suit2 and S2-LM7AA lines. As with occludin, differences in fibronectin expression imparted by ST6Gal-I were eliminated by erlotinib treatment in the Suit2 line (Fig. 7*D*). In S2-LM7AA cells, ST6Gal-I-dependent differences in fibronectin expression were diminished, but not eliminated, by erlotinib. The combined results in Figs. 6 and 7 support the hypothesis that the mesenchymal phenotype of cells with high ST6Gal-I expression may be driven, at least in part, by the sialylation of EGFR.

## Discussion

An enrichment in tumor cell sialylation has long been implicated in carcinogenesis (39, 40, 41, 42). In early studies, enzymatic removal of surface sialic acids blocked tumor cell metastasis in splenic injection models (43). More recently, genetic approaches have been employed to modulate the expression of specific sialyltransferases in animal models, enabling a more mechanistic view of the link between receptor sialylation and malignancy. In a study by Varki’s group, deletion of the *St6gal1* gene in the murine PyMT breast cancer model caused tumor cells to acquire a more differentiated phenotype (44), consistent with the concept that ST6Gal-I confers progenitor-like characteristics. In other studies, our group developed a transgenic mouse with Cre-driven intestinal-specific ST6Gal-I expression and demonstrated that high ST6Gal-I expression promoted colon tumorigenesis in the azoxymethane/dextran sodium sulfate (AOM/DSS) chemical carcinogenesis model (3).

Although substantial evidence points to a protumorigenic function for ST6Gal-I, the mechanisms by which ST6Gal-I regulates tumor cell behavior remain incompletely understood. Results herein describe a role for ST6Gal-I in EMT. RNA-Seq analyses indicated that the Suit2-derived metastatic subclones, S2-013 and S2-LM7AA, had elevated endogenous ST6Gal-I as well as enriched activation of networks associated with EMT, stemness, and cell motility, as compared with the poorly metastatic, parental Suit2 line. Significantly, forced expression of ST6Gal-I in Suit2 cells was sufficient to upregulate EMT, stemness, and cell motility pathways. In line with RNA-Seq results, immunoblotting and qRT-PCR experiments showed that forced expression of ST6Gal-I in parental Suit2 cells increased the expression of mesenchymal markers (snail, N-cadherin, fibronectin) while repressing epithelial markers (E-cadherin, occludin). ST6Gal-I overexpression also promoted cell invasion. Conversely, knockdown of ST6Gal-I in the metastatic S2-LM7AA and S2-013 lines suppressed expression of mesenchymal markers and cell invasiveness while upregulating epithelial markers. These data suggest a causal role for ST6Gal-I in driving EMT.

Although ST6Gal-I has been reported to promote TGFβ-dependent EMT (35), the contribution of ST6Gal-I to EGFR-mediated EMT has not previously been addressed. EGFR is upregulated in approximately 85% of patients with PDAC (45), and hence, the EGFR inhibitor, erlotinib, has been approved by the US Food and Drug Administration to treat PDAC in combination with gemcitabine (46). Consistent with our prior studies showing α2-6 sialylation-dependent EGFR activation in other cancer cell lines (25), ST6Gal-I OE in Suit2 cells increased the α2-6 sialylation and basal activation of EGFR, whereas ST6Gal-I KD in S2-013 and S2-LM7AA diminished EGFR sialylation and activation. Notably, treatment of the Suit2 and S2-LM7AA lines with erlotinib eliminated sialylation-dependent differences in EGFR activation and neutralized ST6Gal-I’s effects on the expression of EMT markers and cell invasiveness. These data lend strong support for the concept that sialylated EGFR contributes to ST6Gal-I’s EMT-promoting activity. However, in the S2-013 line, shC cells maintained markedly higher levels of EGFR activation than KD cells in the presence of erlotinib, for reasons currently unclear. Moreover, erlotinib had little effect on cell invasiveness. We postulate that, in the S2-013 cell model, either the degree of sialylation-dependent EGFR activation in erlotinib-treated shC cells was sufficient to sustain cell invasiveness or, alternatively, the differential sialylation of other ST6Gal-I substrates may have compensated for EGFR in promoting invasion. For example, ST6Gal-I substrates such as the β1 integrin (22, 47) may be an important mediator of invasion in the S2-013 line. Of note, distinct methods were used during the initial generation of the S2-013 and S2-LM7AA subclones. The S2-013 line was developed by first growing cells in soft agar, a procedure that enriches for pathways enabling anchorage-independent cell growth, a process related to integrin signaling. This clone was then injected subcutaneously into the flank to generate primary tumors prior to the collection of metastatic cells. In contrast, the methods used to isolate the S2-LM7AA line more closely mirror the native metastatic process. Parental Suit2 cells were injected into the pancreas, recapitulating the appropriate organ microenvironment, and metastatic cells were then harvested from the liver. The distinct approaches used to generate the S2-013 and S2-LM7AA subclones could have influenced which surface receptors are dominant in *in vitro* invasion assays. Although further studies will be needed to understand the effects of EGFR inhibitors on the S2-013 line, it is noteworthy that in all three cell models (Suit2, S2-013, and S2-LM7AA) ST6Gal-I activity consistently promoted EGFR activation, expression of mesenchymal markers, and cell invasion, confirming the importance of receptor sialylation in EMT.

In summary, results from this investigation add to an accumulating body of literature highlighting a role for ST6Gal-I in endowing tumor cells with more stem-like, mesenchymal characteristics. The transition from an epithelial to mesenchymal phenotype is central to the acquisition of tumor metastatic potential, and thus, elucidating mechanisms underlying EMT is key to the development of more effective cancer treatments. Furthermore, in addition to carcinogenesis, EMT is an essential process in normal human development, as well as wound healing. Although intensive research efforts have focused on EMT, the functional involvement of cell surface sialylation (and more generally, glycosylation) in EMT remains underinvestigated. Our results showing that ST6Gal-I promotes EMT, combined with prior studies by others, underscore the need for further research aimed at delineating sialylation-dependent mechanisms that regulate the phenotype of both normal and malignant cells.

## Experimental procedures

### RNA-sequencing and data analysis

#### RNA preparation and sequencing

RNA was extracted from S2-013, S2-LM7AA, Suit2 EV, and Suit2 OE cells using the Qiagen RNeasy Mini Kit according to the vendor protocol (Catalog # 74104).

RNA concentration was quantified and submitted to the Hugh Kaul Genomics Core at UAB for bulk RNA sequencing. Three biological replicates were generated for each of the four cell lines. mRNA-sequencing was performed on the Illumina NextSeq500 as described by the manufacturer (Illumina Inc, San Diego, CA, USA). Briefly, RNA quality was assessed using the Agilent 2100 Bioanalyzer. RNA with an RNA Integrity Number of ≥7.0 was used for sequencing library preparation. RNA passing quality control was converted to a sequencing ready library using the NEBNext Ultra II Directional RNA library kit as per the manufacturer’s instructions (NEB, Ipswich, MA, USA). The cDNA libraries were quantitated using qPCR in a Roche LightCycler 480 with the Kapa Biosystems kit for Illumina library quantitation (Kapa Biosystems, Woburn, MA, USA) prior to cluster generation. Cluster generation was performed according to the manufacturer’s recommendations for onboard clustering (Illumina, San Diego, CA, USA). We generated between 30 and 35 million paired end 75-bp sequencing reads per sample for gene level abundance.

#### Quantification of gene expression and differential expression

Prealignment quality assessments of the raw fastq sequences were carried out using FastQC (version 0.11.7) (http://www.bioinformatics.babraham.ac.uk/projects/fastqc, accessed September 1, 2020). STAR (version 2.7.3a) was used to align the raw RNA-seq fastq reads to the human reference genome (GRCh38 p13 Release 32) from Gencode using parameters --outReadsUnmapped Fastx; --outSAMtype BAM SortedByCoordinate; --outSAMattributes All (48). Following alignment, HTSeq-count (version 0.11.3) was used to count the number of reads mapping to each gene using parameters -r pos; -t exon; -i gene_id; -a 10; -s no; -f bam (49). Normalization and differential expression were then applied to the count files using DESeq2 (version 1.26.0) using default parameters in their vignette (50). RNA-Seq data are available under NCBI GEO (Accession # GSE158527).

#### Ingenuity Pathway Analysis

IPA Biological Functions and Canonical Pathways were used, along with the Upstream Regulator module (51). For generating networks, a data set containing gene identifiers and corresponding expression values was uploaded into IPA (https://digitalinsights.qiagen.com/products-overview/discovery-insights-portfolio/analysis-and-visualization/qiagen-ipa/, accessed September 1, 2020). Each identifier was mapped to its corresponding object in Ingenuity’s Knowledge Base. A fold change cutoff of ±2 and *p*-value < 0.05 was set to identify molecules whose expression was significantly differentially regulated. These molecules, called Network Eligible molecules, were overlaid onto a global molecular network developed from information contained in Ingenuity’s Knowledge Base. Networks of Network Eligible Molecules were then algorithmically generated based on their connectivity. The Functional Analysis identified the biological functions and/or diseases that were most significant to the entire data set. Molecules from the dataset that met the fold change cutoff of ±2 and *p*-value < 0.05 and were associated with biological functions and/or diseases in Ingenuity’s Knowledge Base were considered for the analysis. Right-tailed Fisher’s exact test was used to calculate a *p*-value determining the probability that each biological function and/or disease assigned to that data set is due to chance alone. For the IPA analyses shown in Table 1, only *Z* scores >1.5 were considered, and at least one of the three cell lines had a *Z* score of ≥2.0.

#### Gene set enrichment analysis

GSEA was performed on the full normalized gene expression data from RNA-Seq experiments, using the GSEA software (version 4.1.0) and the Molecular Signature Database (MSigDB) (version 7.1) (52, 53). The default parameters for GSEA were used except that the permute parameter was set to the gene set. The MSigDB (version 7.1) data sets used include the hallmark curated gene set comprising 50 gene sets (53). For the GSEA plots shown in Figures 1 and 2, the normalized enrichment score and the false discovery rate are provided in the figure legends. The normalized enrichment score is the enrichment score for the gene set after it has been normalized across analyzed gene sets (52). The false discovery rate q-value is the estimated probability that the normalized enrichment score represents a false-positive finding (52).

### Cell culture

Suit2 parental and S2-013 cells were donated by Dr Michael A. Hollingsworth at the University of Nebraska (Omaha, NE, USA). The S2-LM7AA subclone was developed by Drs Lacey McNally and Donald Buchsbaum at the University of Alabama at Birmingham (Birmingham, AL, USA). Suit2 parental and metastatic subclones were maintained in RPMI medium containing 10% fetal bovine serum (FBS) and 1% antibiotic/antimycotic supplements (Gibco). Suit2 parental cells were transduced with either ST6Gal-I overexpression vector (Genecopoeia, cat # LPP-M0351-Lv105-200-5) or empty vector (Sigma) using lentivirus. Metastatic cell lines were transduced with lentivirus encoding shRNA targeting ST6Gal-I (Sigma, cat # TRCN00000035432, sequence CCGGCGTGTGCTACTACTACCAGAACTCGAGTTCTGGTAGTAGTAGC

ACACGTTTTTG), whereas control lines were developed using nontargeting lentivirus bearing shRNA against GFP (Sigma, cat. # SHC002V). Following transduction, stable polyclonal populations were generated by selection with puromycin. Successful overexpression or knockdown was confirmed by immunoblotting using anti-ST6Gal-I goat polyclonal antibody (R&D Systems, cat.# AF5924).

### Immunoblotting

Cells were lysed using radioimmune precipitation assay buffer supplemented with protease and phosphatase inhibitors (Sigma). Total protein concentration was measured using BCA (Pierce). Samples were resolved by SDS-PAGE and transferred to polyvinylidene difluoride membranes. Following transfer, membranes were blocked with 5% nonfat dry milk in tris-buffered saline containing 0.1% Tween20. Immunoblots were probed with antibodies to ST6Gal-I (R&D Systems, cat.# AF5924), pEGFR (pY-1068, Cell Signaling Technology, cat.# 3777), total EGFR (Cell Signaling Technology, cat.# 4267), slug (Cell Signaling Technology, cat.# 9585), snail (Cell Signaling Technology, cat.# 3879), E-cadherin (Cell Signaling Technology, cat.# 3195), and N-cadherin (Cell Signaling Technology, cat.#13116). Protein loading was verified using anti-β-actin (Abcam, cat.# ab20272) or anti-β-tubulin (Abcam, cat.# ab21058). Membranes were incubated with horseradish peroxidase-coupled secondary antibodies (anti-rabbit IgG, Cell Signaling Technology; anti-goat IgG, Santa Cruz Biotechnology), and protein was detected by enhanced chemiluminescence (Pierce).

### SNA Pull-down

Cell lysate, 250 μg, was incubated with 150 μg of SNA-agarose (Vector Labs, cat.# AL-1303). Samples were incubated overnight at 4 °C on a rotator. α2-6 Sialylated proteins were precipitated with centrifugation and washed with ice-cold PBS three times. Precipitates were resolved by SDS-PAGE and immunoblotted for EGFR as described above.

## qRT-PCR

RNA was extracted using the Ambion RNA Extraction Kit (Life Technologies) in accordance with the manufacturer’s protocol. Total RNA concentration was measured, and cDNA was synthesized using M-MLV reverse transcriptase (Promega). qRT-PCR samples were generated using TaqMan Fast Advanced Master Mix (Thermo). Primers for occludin (Hs05465837_g1) fibronectin (Hs01549976_m1), and ST6Gal-I (Hs00949382_m1) were acquired from Applied Biosystems. Data were normalized to GAPDH (Applied Biosystems, Hs02786624_g1), and significance was determined as *p* < 0.05 using a Student’s *t* test from at least three independent experiments, with each independent experiment performed in triplicate.

### Immunocytochemistry

Cells were plated in 24-well plates on sterile glass coverslips with 50,000 cells per well and allowed to attach overnight. Cells were washed with PBS, fixed using 4% paraformaldehyde, and then incubated with E-cadherin antibody (Cell Signaling Technology, cat.# 3195) at a 1:100 dilution at 4 °C overnight. Following incubation with primary antibody, slides were incubated with an anti-rabbit secondary antibody conjugated to Alexa Fluor 488 (Invitrogen, cat.# A32731) at a 1:400 dilution for 1 h at room temperature. Cells were then counterstained with Hoescht nuclear stain at a 1:10,000 dilution for 10 s. The coverslips were mounted on slides using Prolong Gold mounting medium (Invitrogen, cat.# P36930). Cells were viewed using NIS elements software at 20× magnification.

### Invasion assays

*In vitro* cell invasion was evaluated using growth factor reduced invasion chambers (Corning, cat.# 354480). Prior to use, invasion chambers were brought to room temperature and matrigel was rehydrated for 2 h at 37 °C using serum-free medium in a humidified tissue culture incubator. Cells were serum deprived (1% FBS-containing medium) for 24 h and then seeded into the upper well of the invasion chamber at a density of 100,000 cells in 500 μl of 1% FBS-containing media. A 5% FBS-containing medium was added to the bottom chamber and used as a chemoattractant for Suit2 and S2-LM7AA cells; a 2.5% FBS-containing medium was used as the chemoattractant for S2-013 cells. Cells were allowed to invade through matrigel for 24 h at 37 °C. After incubation, the medium containing noninvaded cells, as well as the matrigel layer, were carefully removed from the upper portion of the chamber. The underside of the transwell membrane, containing the adherent cells that had successfully invaded the matrigel, was fixed with 4% paraformaldehyde and stained with 0.5% (w/v) crystal violet. Crystal violet was dried overnight and then solubilized using a 10% acetic acid solution. Absorbance of the solution was quantified at 590 nm on a Biotek plate reader. Absorbance was normalized to that of controls (cells plated in chambers with no chemoattractant). All invasion assays were conducted at least three independent times, and each independent experiment was performed in triplicate. Significance was determined as *p* < 0.05 using a Student’s *t* test.

### Erlotinib treatment

For immunoblotting and qRT-PCR experiments using erlotinib, cells were serum deprived for 2 h using a 1% FBS-containing medium before treatment with 20 μM erlotinib for 24 h. Lysates were then collected for protein or RNA quantification as described above. Invasion assays using erlotinib-treated cells were performed as described above. Prior to plating in invasion chambers, detached cells were incubated with 20 μM of erlotinib in suspension on a rotator at 37 °C for 2 h. Cells were then plated, with inhibitor, in invasion chambers, and the invasion assay was completed as described previously.

## Data availability

All of the data described in this report are contained within the manuscript or supplementary figures, with the exception of the RNA-Seq results. The RNA-Seq data are available under NCBI GEO Accession# GSE158527.

## Conflict of interest

The authors declare that they have no conflicts of interest with the contents of this article.
