# Supplementary material for: Glycosyltransferase ST6Gal-I promotes the epithelial to mesenchymal transition in pancreatic cancer cells
Source: J Biol Chem. 2020 Nov 23;296:100034. doi: 10.1074/jbc.RA120.014126 (PMC7949065; doi:10.1074/jbc.RA120.014126)
Supplement: Tables S1-S4 [file mmc1.pdf]

Supplementary Results

RNA-Seq was conducted to compare gene expression in parental Suit2 cells with two isogenic, metastatic Suit2 subclones, S2-013 and S2-LM7AA. In addition, ST6Gal-I was overexpressed (OE) in Suit2 parental cells, and gene expression in OE cells compared with control, empty vector (EV)-transduced Suit2 cells. Data from these experiments are included in the following Supplementary Tables:

Supplementary Table 1. Genes altered in S2-013 and S2-LM7AA cells relative to Suit2 EV cells.
This table lists the top 50 upregulated and downregulated genes in the two metastatic subclones compared to parental, poorly-metastatic Suit2 cells.

Supplementary Table 2. IPA Upstream Regulators altered in S2-013 and S2-LM7AA cells relative to Suit2 EV cells.
In this table, the Ingenuity Pathway Analysis (IPA) Upstream Regulators module was used to identify the pathways either activated or inhibited in the two metastatic subclones as compared with parental Suit2 cells.

Supplementary Table 3. Genes altered in Suit2 OE versus Suit2 EV cells
This table lists the top 50 upregulated and downregulated genes in Suit2 OE cells as compared to Suit2 EV cells.

Supplementary Table 4. IPA Upstream Regulators altered in Suit2 OE relative to Suit2 EV cells.
In this table, the IPA Upstream Regulators module was used to identify the pathways either activated or inhibited in Suit2 OE cells as compared to Suit2 EV cells.

Supplementary Table 1. Genes altered in S2-013 and S2-LM7AA relative to Suit2 EV cells

A Top 50 upregulated genes

S2-013		S2-LM7AA	
gene name	Z Score	gene name	Z Score
CHST9	4.9791565	CHRM3	4.848015
BOC	4.967989	EPHA7	4.834556
TRABD2B	4.9650354	SETBP1	4.8167334
CRMP1	4.9584484	GJA1	4.7665424
SPOCK1	4.953624	LDLRAD4	4.741674
ERBB4	4.953421	P2RY1	4.71017
SLC17A3	4.9468355	WFDC1	4.6820936
RGS4	4.9465475	AUTS2	4.635952
EVC	4.943861	PLAT	4.589742
EPHA7	4.9315243	KCNJ15	4.5756273
PTGER2	4.9291673	CALD1	4.5452437
ISM1	4.918633	LRIG1	4.5425453
PCYT1B	4.9124303	GRID1	4.493754
ENOX1	4.9119964	KCNJ12	4.4503117
DNAJC5B	4.906601	KCNJ4	4.3913965
TNFAIP6	4.899787	CAMK4	4.3471484
LNCAROD	4.8977523	HLX	4.339615
KCNJ15	4.890724	FEZ1	4.3220263
WFDC1	4.8895035	BVES	4.2801723
CRYAB	4.880079	NTF3	4.2795386
EPHB1	4.8786187	TMEFF2	4.273749
HES7	4.873289	DSCAML1	4.2371936
SEMA6A	4.8631697	PDGFD	4.2251496
VAV3	4.8450937	VCAM1	4.2105227
NTNG2	4.834516	ADGRA2	4.192516
AFF3	4.8277216	ASB9	4.1669703
FAM155A	4.826208	NACAD	4.1570516
SEMA6B	4.81453	CD226	4.144238
MIOX	4.8108425	LPAR3	4.1395035
CALD1	4.8006387	ISM1	4.121965
PRDM16	4.800318	HLA-DPB1	4.108773
GPC4	4.7849913	LYPD6	4.085726
SMIM32	4.7691774	CNIH3	4.0844517
PLCB4	4.7635036	TIMP3	4.063056
HLF	4.752887	LZTS1	4.0617776
ALPK2	4.743556	SGK1	4.0599556
TSPAN7	4.7234907	ANGPTL2	4.0381
INHBB	4.715522	RNVU1-4	4.029565
PRR16	4.70858	P3H3	4.0244584
STK32B	4.703925	SRPX	4.009984
DCDC2	4.7028785	TSHZ3	4.007069
HRK	4.701378	TRIM22	3.9996552
DPF3	4.6949487	OSBPL6	3.9904444
SLC16A7	4.6945863	NLGN1	3.987834
MITF	4.694077	CLDN16	3.9798505
FXYD2	4.6820965	PAK3	3.9570365
SLC6A15	4.679244	NKAIN	3.9442637
AQP1	4.6703873	SUSD5	3.9180527
ADGRA2	4.663525	FLT1	3.9056764
OTOP1	4.663312	AK5	3.898411

B Top 50 downregulated genes

S2-013		S2-LM7AA	
gene name	Z Score	gene name	Z Score
C4BPB	-4.9913206	GALNT3	-4.851432
CDH2	-4.9894357	CEACAM6	-4.8127456
TMEM176A	-4.989257	PHACTR3	-4.7663064
CCDC152	-4.981356	MAL2	-4.7495804
UGT1A8	-4.978773	SCEL	-4.73677
ADH1C	-4.978284	BSPRY	-4.709945
TBX2	-4.9756083	MYO1D	-4.6571813
TMEM176B	-4.97384	FGF19	-4.6166573
CYP2C18	-4.9592485	STX19	-4.616506
TSPAN8	-4.954827	DAPP1	-4.588257
HSD17B2	-4.9410048	EPHB6	-4.573664
TM4SF20	-4.928785	PAG1	-4.521394
NRIP3	-4.9246254	RAB25	-4.390008
CLRN3	-4.919323	ZNF334	-4.375597
SERPINB5	-4.906589	SPNS2	-4.3255653
SERPINE2	-4.9061775	TMEM30B	-4.3226357
GASK1B	-4.883624	WNT10A	-4.288116
MUC13	-4.8676414	FAM131B	-4.2616997
HHLA2	-4.8645015	ESRP1	-4.205865
CLCN1	-4.8588605	BHLHE41	-4.1997476
AGR2	-4.8586965	GPX2	-4.1956625
GPA33	-4.8570194	FOXJ1	-4.177557
ZNF334	-4.838888	EPN3	-4.1530743
RASGEF1B	-4.824592	KDF1	-4.1398654
AKR1C2	-4.8205986	CBLC	-4.133392
IKZF2	-4.7992053	ITGB6	-4.1187105
UGT1A10	-4.7964425	AKR1B10	-4.114817
PKIA	-4.778929	MARVELD3	-4.099542
CEACAM6	-4.7645407	MPP7	-4.073924
KCNJ3	-4.740252	CDH1	-4.070056
KIAA0040	-4.7266226	FAM83F	-4.068028
STEAP1	-4.7240286	FAM83A	-4.055757
FGF19	-4.692759	TRBC2	-3.991412
RASSF9	-4.660843	TMPRSS13	-3.9877846
ANPEP	-4.647873	CYP2C18	-3.9834173
SNAI2	-4.641878	RBBP8NL	-3.9809165
HAS2	-4.6326146	ANO1	-3.9807398
SPINK7	-4.624757	FUT2	-3.9651875
VSTM2L	-4.622706	KCNJ3	-3.949789
MOB3B	-4.6150417	MPZL2	-3.947292
XYLT1	-4.6099906	MACC1	-3.942559
CGREF1	-4.601841	ENPP5	-3.9124942
ADH6	-4.597636	ANKRD22	-3.8937342
CYP4F3	-4.5905004	CYP2C9	-3.887514
SLC38A11	-4.58188	NCMAP	-3.8839972
B3GALT1	-4.563985	SPRR1B	-3.8657577
MXRA5	-4.5547056	OVOL1	-3.855813
PLA2R1	-4.552125	P2RY2	-3.8537807
OMP	-4.5512743	RAB11FIP4	-3.8528874
CXCL1	-4.5478554	TSPAN2	-3.8372097

Supplementary Table 2. IPA Upstream Regulators altered in S2-013 and S2-LM7AA relative to Suit2 EV cells

A Upstream Regulators with predicted activation

S2-013	
Regulator	Z Score
DSCAM	4.122
TEAD4	4.086
TEAD1	3.979
NRG1	3.729
CCR2	3.687
SIM1	3.637
TEAD3	3.578
ARNT2	3.569
NSUN6	3.5
MRTFB	3.466
DSCAML1	3.452
Tgf beta	3.452
CBX5	3.317
TEAD2	3.3
TGFB1	3.141
MAPK1	3.046
BDNF	2.943
LLGL2	2.887
glucocorticoid	2.863
NOTCH1	2.842
SMAD3	2.796
DLX5	2.789
Wnt	2.739
Tcf7	2.709
WWTR1	2.697
HIF1A	2.695
BMP6	2.664
TFAP2A	2.59
D-glucose	2.569
LATS2	2.534
CTNNB1	2.493
KMT2D	2.468
FBXO32	2.467
TAZ	2.467
F2	2.45
EDNRA	2.449
ARRB1	2.433
GLI1	2.433
WBP2	2.419
NFIC	2.414
VDR	2.385
STK40	2.384
NCOA4	2.383
PLAG1	2.377
TP53	2.35
SNAI2	2.343
STAT5B	2.336
MET	2.325
JAG1	2.315
BRD4	2.273
CIITA	2.27
WNT1	2.245
Growth hormone	2.226
DLX6	2.219
TEK	2.219
MMP7	2.213
KDM3A	2.197
NMNAT1	2.197
IL1R2	2.186
ROCK1	2.178
PPP1CA	2.176
TET3	2.176
APLN	2.164
TRIM59	2.157
HRG	2.138
NORAD	2.138
IL10	2.115
GLI2	2.103
CSF1	2.089
ERBB3	2.084
LEF1	2.083
ROR1	2.074
JAK1/2	2.065
EBI3	2.06
FOXC2	2.048
EGR2	2.025
Il3	2.025
EREG	2.012
HCAR1	2
SMC3	2

S2-LM7AA	
Regulator	Z Score
TGFB1	4.557
MRTFB	4.182
Tgf beta	4.016
NRG1	3.789
MRTFA	3.491
F2	3.441
WNT3A	3.388
EDN1	3.378
LLGL2	3.317
CCR2	3.111
SMARCA4	3.071
CTNNB1	3.043
SHH	2.95
BDNF	2.914
NSUN6	2.887
EPAS1	2.881
D-glucose	2.861
MAPK1	2.852
FGF8	2.815
ROR1	2.813
SMAD1	2.805
SMAD3	2.791
AGER	2.787
GLI1	2.787
NORAD	2.714
TWIST1	2.709
DSCAM	2.683
ROCK1	2.621
ATF2	2.615
IPMK	2.611
Interferon α	2.576
RUNX1	2.563
IgG	2.542
TEAD2	2.53
cyclic AMP	2.528
ETS1	2.523
NOTCH3	2.513
F3	2.466
MTPN	2.459
SP1	2.435
CBX5	2.414
GDF9	2.392
FEV	2.357
IL6	2.34
ARNT2	2.335
CYP1B1	2.333
RETNLB	2.331
LEF1	2.314
progesterone	2.311
TEAD1	2.309
CG	2.268
MAPK14	2.268
IFNAR1	2.249
KDM3A	2.241
HRG	2.236
JAG1	2.223
IFNA2	2.212
WBP2	2.212
EGF	2.207
GAB2	2.201
Fgf	2.2
PDGFC	2.198
Tnf receptor	2.19
TGFBR1	2.181
BRD4	2.178
Secretase gamma	2.177
MYOD1	2.166
RAF1	2.163
RAC1	2.152
TEAD4	2.137
VCAN	2.126
CLEC4G	2.121
CYP1A2	2.121
TEAD3	2.111
PTK2	2.103
Ap1	2.086
ZEB1	2.06
WWTR1	2.041
SIM1	2.038
TCF7L2	2.01
βcatenin/TCF	2
Endothelin	2
RXFP2	2

B Upstream Regulators with predicted inhibition

S2-013	
Regulator	Z Score
RUNX3	-3.665
EGLN	-3.344
PTPN1	-3.293
FAS	-3.132
HRAS	-3.016
NR1H4	-2.999
OGA	-2.898
IKZF3	-2.605
SFTPA1	-2.578
GFI1	-2.505
CXCR3	-2.425
IL17R	-2.414
TNFSF12	-2.393
TRADD	-2.345
FOSL1	-2.286
NCOR1	-2.236
PTPRE	-2.236
SAV1	-2.236
SPDEF	-2.226
SFRP1	-2.214
RORC	-2.213
KMT5B	-2.2
LTF	-2.2
NFKBIZ	-2.193
RIPK2	-2.134
IL36A	-2.072
DL-fructose	-2.046
FGF1	-2.039
DICER1	-2.027
CLOCK	-2.024
GATA6	-2.006
Atrial Natri.Peptide	-2
CISH	-2
FAF1	-2
LGALS8	-2
MEDAG	-2

S2-LM7AA	
Regulator	Z Score
RUNX3	-3.969
SPDEF	-3.771
PKD1	-3.573
Alpha catenin	-3.386
estrogen receptor	-3.375
FGF1	-3.347
FAS	-3.231
CR1L	-3.121
ALDH1A2	-2.985
SMAD7	-2.75
GFI1	-2.693
DICER1	-2.649
GAPDH	-2.621
FBN1	-2.607
REST	-2.414
HNF1A	-2.391
SOCS1	-2.311
CIP2A	-2.309
KRAS	-2.3
ABCB4	-2.294
Atrial Natri.Peptide	-2.236
SPINT1	-2.224
NOSTRIN	-2.208
MED16	-2.205
TNFRSF1A	-2.191
HNF1B	-2.176
DKK1	-2.172
FOXA3	-2.122
GPX1	-2.07
TAF4	-2.007
CHD1	-2
GLIS2	-2
SFRP4	-2

Supplementary Table 3. Genes altered in Suit2 OE versus Suit2 EV cells

A Top 50 upregulated genes

Suit2 OE vs. Suit EV	
**gene name**	**Z Score**
BASP1	4.9322567
STK32B	4.8835506
EPHB1	4.7745857
ST6GAL1	4.7094207
KCNMA1	4.5498943
KCNJ15	4.4093223
PDGFD	4.2174225
CALD1	4.172452
WFDC1	3.9941797
P2RY1	3.9835193
ANXA8L1	3.9461062
SLC16A7	3.8598228
DPF3	3.8140402
ALPK2	3.775378
ILDR2	3.7584732
SEMA6B	3.7221954
SNCG	3.6903024
PGBD5	3.6679268
DPYSL5	3.6629093
FXYD2	3.6378546
WNT5A	3.6239634
IGFBP2	3.5305097
NEURL1	3.486795
AQP1	3.480325
SPOCK1	3.467746
BTNL8	3.4442382
FRMD3	3.4297755
DRAXIN	3.4053168
NUGGC	3.4053168
SLC6A15	3.3929842
TARID	3.3746426
AFF3	3.352433
RGS4	3.3237362
CCDC80	3.2820559
SPP1	3.2807908
SFTPD	3.2591472
CAMK4	3.2503872
HES7	3.2448862
SRPX	3.2286468
NLGN1	3.199847
LPAR3	3.1771877
TRIM9	3.162713
LOXL2	3.152729
LRIG1	3.1396716
SMIM32	3.138023
DCLK2	3.1370163
ANTXR1	3.127695
TNC	3.1158962
MITF	3.0908923
AUTS2	3.0765173

B Top 50 downregulated genes

Suit2 OE vs. Suit2 EV	
**gene name**	**Z Score**
GASK1B	-3.3527489
TCIM	-2.9040418
FCER1G	-2.8452768
ANGPTL7	-2.7785919
HHLA2	-2.7483401
ANXA10	-2.3554127
PRR35	-2.348888
CCDC175	-2.2943113
CYP2C18	-2.2828605
DHRS9	-2.250698
CIDEC	-2.1772063
SLC16A9	-2.1759825
AKR1B10	-2.0901911
SIDT1	-2.0725164
MMP1	-2.0614552
ZC3H11A	-2.0372212
FOXF1	-2.028866
BAMBI	-2.0213413
AKR1B15	-2.0111032
WNT7A	-2.007517
SULT1C2	-1.9560361
CLCN1	-1.9129947
GUCY1A1	-1.9120764
RGS2	-1.9100358
SNORA59B	-1.9020985
PKIA	-1.8948313
ERFL	-1.8901143
KRT17	-1.8871663
CYP2C9	-1.8866144
SLC9A2	-1.8684675
DPP4	-1.8655809
XK	-1.8138207
CEACAM6	-1.8080875
LRRN4CL	-1.797032
SNORA71B	-1.7757243
LNCAROD	-1.7651157
ABCC2	-1.750571
P2RY2	-1.749371
CES4A	-1.7472205
SPRR1B	-1.7442523
AGMAT	-1.6901253
IL1RL1	-1.6725954
H3C8	-1.6652617
S100P	-1.6648424
EVA1A	-1.648896
TFF1	-1.6450198
DDX4	-1.6357026
RAET1L	-1.6357026
CELA2B	-1.6357026
KYNU	-1.6319162

Supplementary Table 4. IPA Upstream Regulators altered in Suit2 OE relative to Suit2 EV cells

A Upstream Regulators with predicted activation

Suit2 OE vs. EV	
**Regulator**	**Z Score**
ARNT2	4
TGFB1	3.943
HIF1A	3.905
NRG1	3.696
TP53	3.68
SMARCA4	3.669
SIM1	3.615
STAT3	3.546
Tgf beta	3.543
NFKBIA	3.537
WNT3A	3.535
KLF4	3.358
SP1	3.327
EHMT1	3.317
DSCAML1	3.3
SMAD3	3.296
F2R	3.265
AGT	3.131
VitaminD3-VDR-RXR	3.127
cyclic AMP	3.105
JAG1	3.086
SMARCB1	3.053
WBP2	3.036
LEF1	3.026
MTPN	2.97
aldosterone	2.962
TGFBR1	2.942
RAF1	2.936
VEGFA	2.935
MRTFB	2.883
FEV	2.828
RETNLB	2.828
MAFB	2.789
F2	2.787
PDGFB	2.777
APLN	2.728
GDF2	2.718
TGM2	2.692
SHH	2.689
DSCAM	2.688
NFkB (complex)	2.671
SGK1	2.646
NORAD	2.646
NSUN6	2.646
LLGL2	2.646
EPAS1	2.64
T3-TR-RXR	2.63
BRD4	2.626
CCL5	2.596

Suit2 OE vs. EV (continued)	
**Regulator**	**Z Score**
NFAT5	2.592
SRC (family)	2.588
BDNF	2.58
Growth hormone	2.469
ZAP70	2.449
CD247	2.449
TEAD4	2.449
KDM3A	2.433
JUN	2.433
ROCK1	2.425
cholesterol	2.395
BMP2	2.381
IL27	2.359
KITLG	2.343
glucocorticoid	2.332
SOX4	2.331
SOX11	2.306
CCN2	2.297
CG	2.287
SMAD4	2.264
AR	2.237
IFNAR1	2.236
TIMP1	2.236
ROR1	2.236
THRB	2.234
ERG	2.228
OTX2	2.216
FGF10	2.213
Complement	2.213
GLI1	2.208
ARID1A	2.2
TAF7L	2.2
RARB	2.197
CEBPB	2.193
FOXC2	2.169
Brd4	2.138
F3	2.135
D-glucose	2.118
TP73	2.096
MRTFA	2.078
IL13	2.068
STAT1	2.038
SMAD1	2.038
CNR1	2.027
P38 MAPK	2.023
HOXB13	2
FHL2	2
vitamin K2	2
HRG	2

B Upstream Regulators with predicted inhibition

Suit2 OE vs. EV	
**Regulator**	**Z Score**
Alpha catenin	-3.894
EGLN	-3.773
ZNF217	-3.162
CBX5	-2.887
IKZF3	-2.828
FFAR3	-2.813
PSMB11	-2.714
SYK	-2.63
FAS	-2.614
SOX1	-2.53
SPDEF	-2.53
DNMT3B	-2.5
EED	-2.449
CR1L	-2.412
DKK1	-2.393
KDM1A	-2.341
KDM3B	-2.236
NPC2	-2.236
GMNN	-2.145
COL18A1	-2.143
REST	-2.135
CDKN1B	-2.132
CIP2A	-2.121
SOX3	-2.111
KRAS	-2.081
LRP1	-2
LATS1	-2
